# The Impact of COVID-19 during Pregnancy on Maternal Hemodynamic Function, Angiogenic Markers and Neonatal Outcome

**DOI:** 10.3390/v16060868

**Published:** 2024-05-29

**Authors:** Nawa Schirwani-Hartl, Lena Tschanun, Pilar Palmrich, Christina Haberl, Nicole Perkmann-Nagele, Herbert Kiss, Angelika Berger, Julia Binder

**Affiliations:** 1 Department of Obstetrics and Gynecology, Division of Obstetrics and Feto-Maternal Medicine, Medical University of Vienna, 1090 Vienna, Austria; nawa.schirwani-hartl@meduniwien.ac.at (N.S.-H.); n01605787@students.meduniwien.ac.at (L.T.); pilar.palmrich@meduniwien.ac.at (P.P.); christina.haberl@meduniwien.ac.at (C.H.); herbert.kiss@meduniwien.ac.at (H.K.); 2 Department of Laboratory Medicine, Medical University of Vienna, 1090 Vienna, Austria; nicole.perkmann-nagele@meduniwien.ac.at; 3 Department of Pediatrics and Adolescent Medicine, Division of Neonatology, Pediatric Intensive Care and Neuropediatrics, Comprehensive Center for Pediatrics, Medical University of Vienna, 1090 Vienna, Austria; angelika.berger@meduniwien.ac.at

**Keywords:** SARS-CoV-2, pregnancy, USCOM, arteriograph, sFlt-1/PlGF, preeclampsia, fetal growth restriction

## Abstract

Infections with severe acute respiratory syndrome coronavirus 2 (SARS-CoV2) in pregnancy are associated with the development of preeclampsia and fetal growth restriction (FGR). Recently, preeclampsia was linked to impaired maternal hemodynamic function. This retrospective study evaluated singleton pregnancies with COVID-19 during pregnancy and healthy pregnant controls matched for gestational age from November 2020 to March 2022. Non-invasive assessment of maternal hemodynamics by continuous wave Doppler ultrasound measurements (USCOM-1A^®^ Monitor) and oscillometric arterial stiffness (Arteriograph) was performed. Overall, 69 pregnant women were included—23 women after COVID-19 during pregnancy and 46 healthy controls. While two women (8.7%) were admitted to the hospital due to COVID-19-related symptoms, none required intensive care unit admission or non-invasive/invasive ventilation. There were no statistically significant differences in the majority of hemodynamic parameters between the two cohorts. The prevalence of FGR was significantly higher in the COVID-19 during pregnancy group (9.5% vs. healthy controls: 0.0%; *p* = 0.036), especially in nulliparous women. No difference in angiogenic markers and neonatal outcomes were observed between pregnant women after COVID-19 and healthy controls. In conclusion, no significant differences in hemodynamic parameters or neonatal outcome were observed in women with COVID-19 during pregnancy. However, an increased prevalence of FGR could be described.

## 1. Introduction

The coronavirus disease 19 (COVID-19) pandemic, caused by the severe acute respiratory syndrome coronavirus 2 (SARS-CoV-2), resulted in significant morbidity and mortality worldwide [1,2,3,4]. Apart from respiratory symptoms [5], SARS-CoV-2 induces a systemic disease via the angiotensin converting enzyme (ACE)-2 receptor, its primary binding site [6,7]. The ACE-2 receptor is—among various other locations—represented in the placenta, decidua and general endothelial cells [8,9]. Pregnant women, due to their immunosuppressive state, constitute a high-risk group for severe COVID-19 [10]. Moreover, SARS-CoV-2 infection during pregnancy has been associated with possible direct and several indirect negative effects for the fetus, including increased risk of stillbirth or preterm delivery [11,12]. Despite the presence of several different vaccines against COVID-19, which are considered safe in pregnancy and are recommended for pregnant women [13], this population is still at risk when infected by SARS-CoV-2 during pregnancy.

Many different cardiovascular alterations have been described in pregnant women with COVID-19 [14]. One case series reported decreased left ventricular ejection fraction and elevated cardiac markers such as pro-brain-type natriuretic peptide (proBNP) in critically ill pregnant women with COVID-19, with a mortality rate of 13% due to cardiac arrhythmia [15]. In another study investigating pregnant women without preexisting cardiovascular disease suffering from severe COVID-19, 22% were reported to have elevated cardiac troponins, 30% elevated NT-proBNP and one third of patients had bradycardia [16]. Moreover, it has been demonstrated that women with SARS-CoV-2 infection during pregnancy have an increased risk of preeclampsia [17]. Importantly, one study showed that the association between preeclampsia and COVID-19 during pregnancy is independent of preexisting conditions and COVID-19 severity [18]. Furthermore, nulliparous women with SARS-CoV-2 infection during pregnancy seem to be at a particularly high risk of preeclampsia compared to multiparous women [18].

However, despite the mounting evidence of a potential connection between SARS-CoV-2 infection during pregnancy and the maternal cardiovascular system, detailed information on changes in maternal hemodynamics and effects on the fetus is still scarce. As a result, the aim of this study was to investigate hemodynamic parameters and angiogenic markers as well as fetal outcome parameters in pregnant women after COVID-19 in pregnancy compared to a control group of healthy pregnant women without COVID-19.

## 2. Materials and Methods

### 2.1. Study Design and Study Participants

This is a retrospective case-control study conducted at the Department of Obstetrics and Feto-Maternal Medicine at the Medical University of Vienna. We included pregnant women after SARS-CoV-2 infection during pregnancy scheduled for hemodynamic assessment between November 2020 and March 2022, as well as a control group of healthy pregnant women undergoing the same hemodynamic assessments between October 2019 and June 2021. Pregnant women and healthy controls were matched in a 1:2 ratio for maternal age and gestational age at the time of hemodynamic measurement.

The inclusion criteria for the COVID-19 during pregnancy cohort were as follows: singleton pregnant women with a history of SARS-CoV-2 infection during any time point in pregnancy and age ≥18 years. The healthy control cohort consisted of healthy pregnant women undergoing hemodynamic assessment as part of the Biobank of the Department of Obstetrics and Feto-Maternal Medicine and included women without any preexisting medical conditions after informed consent (2306/2020).

Clinical characteristics including maternal age, height and body weight, body mass index (BMI), mode of conception, gestational age (GA) at the time of hemodynamic measurement, parity, presence of cardiovascular disease, lung disease, diabetes mellitus, hematological disease, liver disease, endocrinological disease, neurological disease, renal disease, smoking and alcohol intake during pregnancy, as well as GA at the time of birth and pregnancy outcome (e.g., mode of birth, live birth, fetal death) were extracted from the obstetric electronic database (Viewpoint 5.6.8.428, Wessling, Germany). Neonatal outcomes included sex, birth weight, fetal growth restriction (FGR) as defined by the 2016 Delphi consensus-based criteria [19], APGAR score at 1, 5 and 10 min after birth and admission to newborn intensive care unit (NICU).

### 2.2. Diagnosis and Definitions of COVID-19

Pregnant women with a history of COVID-19 during pregnancy, who underwent hemodynamic assessment at the Department of Obstetrics and Feto-Maternal Medicine at the Medical University of Vienna were included in this study. The date of the first positive PCR test, symptoms after SARS-CoV-2 infection and COVID-19 outcomes, such as hospital admission, ICU admission and ventilation, were recorded. COVID-19 symptoms including for example fever, nausea, headache, myalgia, vertigo and fatigue were assessed using a specifically designed questionnaire, which was performed at the time of hemodynamic assessment.

### 2.3. Hemodynamic Measurements

Non-invasive hemodynamic assessment included continuous wave Doppler ultrasound using the Ultrasonic Cardiac Output Monitor (USCOM-1A, Uscom Ltd., Sydney, Australia). All measurements were conducted by operators with according training in using the device. While the patient was lying in a slightly tilted position, the USCOM-1A probe was placed on the suprasternal notch to measure velocity time integrals (VTIs) of transaortic blood flow at the left ventricular outflow tract [20]. The device uses an algorithm, which correlates the outflow tract diameter with the height of the patient to produce stroke volume (SV) and cardiac output (CO) as well as a thorough hemodynamic calculation, including heart rate (HR) and systemic vascular resistance (SVR) among other parameters [20].

Oscillometric arterial stiffness assessment (Arteriograph, TensioMed, Budapest, Hungary) was conducted according to a previously described protocol [21]. A single non-invasive blood pressure cuff was positioned on the women’s left upper arm [22]. Pressure receptors in the cuff are influenced by blood pressure variations and transferred to a computer via an infrared port. Systolic and diastolic blood pressure are measured with the first cuff inflation. Subsequently, a second inflation at 35 mmHg over the systolic blood pressure is conducted and the pulse wave configuration is documented. This creates two systolic peaks. While the first peak (P1) is generated by blood volume ejection from the left ventricle into the aorta, the second peak (P2) is generated by the reflected wave from the periphery. The Brachial augmentation index (Ax), which is a marker for brachial arterial stiffness [23], is calculated by subtracting the second peak (P2) from the first peak (P1), dividing the result by the pulse pressure (PP) and multiplying the result of this by 100. The Aortic Ax is derived from its relationship with Brachial Ax; the exact formula has been previously described [24]. Aortic pulse wave velocity (PWVao) is calculated by dividing the jugular fossa-to-symphysis distance by the return time (RT) divided by 2. RT is related to the stiffness of the aorta and calculated by the difference between P1 and P2. The results were obtained using the regularly updated Arteriograph software version.

All measurements were conducted while the patient was lying slightly tilted to the left side.

### 2.4. Laboratory Measurements

Serum soluble Fms-like tyrosine kinase-1 (sFlt-1), placental-like growth factor (PlGF) and their ratio were obtained in pregnant women with COVID-19 during pregnancy and healthy pregnant controls at time of the hemodynamic assessment and analyzed by using an automated immune analyzer by Roche Diagnostics, Mannheim, Germany.

### 2.5. Statistical Analysis

Continuous data were reported as mean ± standard deviation or median and interquartile ranges (IQR), as appropriate. Collected categorical parameters were presented as numbers (n) and percentages (%) of patients with the characteristic of interest. For comparing continuous variables between two groups, an unpaired t-test was applied. In order to compare continuous variables without normal distribution between the two groups, a Mann–Whitney U test was performed. For group comparisons of categorical variables, Pearson’s Chi-squared test was used. IBM SPSS 25.0 statistical software (IBM, Armonk, NY, USA) and GraphPad Prism (Graphpad Software, version 8.0 La Jolla, CA, USA) were utilized for statistical analysis. For statistical significance, a two-sided *p*-value of *p* < 0.050 was applied.

## 3. Results

### 3.1. Study Population

Overall, 23 pregnant women with COVID-19 during pregnancy, as well as 46 healthy pregnant women were included in this study. Details on patient characteristics are provided in Table 1.

The median maternal age was 32.2 (interquartile range [IQR] 28.8–35.7) years in the COVID-19 during pregnancy cohort and 31.9 (IQR 29.3–35.6) years in the healthy pregnant control cohort (*p* = 0.932). All included women had singleton pregnancies. The mean gestational age at the time of hemodynamic measurement was 25.5 ± 5.6 gestational weeks in the COVID-19 during pregnancy cohort and 24.0 ± 5.9 gestational weeks in the healthy control cohort (*p* = 0.324).

### 3.2. Symptoms and Outcomes of COVID-19

Table 2 provides detailed information on the symptoms after SARS-CoV-2 infection in the COVID-19 during pregnancy cohort.

Information on COVID-19 symptoms were available in 17/23 (73.9%) of the included pregnant women. The most frequent symptoms were loss of smell/taste (76.5%), rhinitis (70.6%), cough (70.6%), headache (64.7%) and vertigo/fatigue (64.7%). Moreover, 41.2% of women in the COVID-19 during pregnancy cohort had fever with a median duration of 3.0 days.

Two women (8.7%) were admitted to the hospital due to their COVID-19 symptoms. However, none of the women in the COVID-19 during pregnancy cohort had to be admitted to the intensive care unit and none required non-invasive or invasive ventilation. In summary, while a number of typical symptoms were frequent in the COVID-19 during pregnancy cohort, the women generally presented with mild courses of the disease.

Included women in the COVID-19 cohort and healthy controls did not develop gestational hypertension, preeclampsia or HELLP syndrome.

### 3.3. Preeclampsia Markers and Hemodynamic Parameters after COVID-19 during Pregnancy

The median sFlt-1/PlGF ratio (COVID-19: median 4.3 [IQR 2.6–5.8] vs. healthy controls: median 3.6 [IQR 1.8–6.3]; *p* = 0.795) did not differ between the two groups and was well within the normal range in the COVID-19 during pregnancy cohort.

As shown in Table 3, there were no statistically significant differences in most hemodynamic parameters between the COVID-19 during pregnancy and the healthy control cohorts. Mean arterial pressure (MAP; COVID-19: 83.0 [IQR 79.0–95.0] mmHg vs. healthy controls: 82.5 [IQR 79.0–87.5] mmHg; *p* = 0.544) and cardiac index (COVID-19: 3.6 [IQR 3.2–4.1] L/min/m^2^ vs. healthy controls: 3.6 [IQR 3.1–4.0] L/min/m^2^; *p* = 0.797) were almost identical in both cohorts. Interestingly, stroke work (COVID-19: 1060.0 [IQR 901–1161.0] mmHg/mL vs. healthy controls: 926.5 [IQR 812.0–1072.0] mmHg/mL; *p* = 0.033) was significantly higher in the COVID-19 during pregnancy cohort.

**Figure 1:**
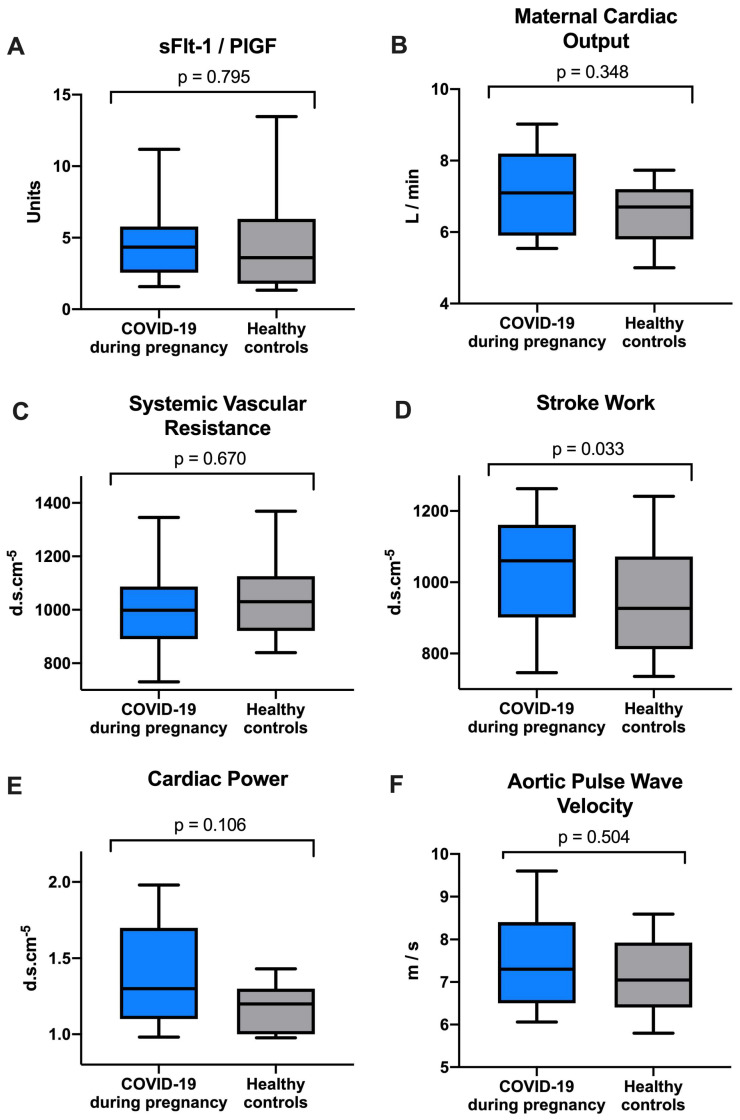
Comparison of (**A**) sFlt-1/PlGF, (**B**) maternal cardiac output, (**C**) systemic vascular resistance, (**D**) stroke work, (**E**) cardiac power and (**F**) aortic pulse wave velocity between women with COVID-19 during pregnancy and healthy controls.

Median cardiac output (COVID-19: 7.1 [IQR 5.9–8.2] L/min vs. healthy controls: 6.7 [IQR 5.8–7.2] L/min; *p* = 0.348), heart rate (COVID-19: 80.0 [IQR 72.0–87.0]/min vs. healthy controls: 75.0 [IQR 69.0–82.0]/min; *p* = 0.443), stroke volume (COVID-19: 92.0 [IQR 81.0–103.0] mL vs. healthy controls: 87.0 [IQR 73.0–96.0] mL; *p* = 0.268) and cardiac power (COVID-19: 1.3 [IQR 1.1–1.7] W vs. healthy controls: 1.2 [IQR 1.0–1.3] W; *p* = 0.106) were numerically higher in the COVID-19 during pregnancy cohort without reaching statistical significance. Median systemic vascular resistance (SVR; COVID-19: 998.0 [IQR 890.0–1087.0] d.s.cm^−5^ vs. healthy controls: 1030.0 [IQR 921.0–1125.5] d.s.cm^−5^; *p* = 0.670), inotropy index (COVID-19: 1.6 [IQR 1.5–1.8] W/m^2^ vs. healthy controls: 1.8 [IQR 1.5–1.8] W/m^2^; *p* = 0.670) and corrected flow time (COVID-19: 370.0 [IQR 348.0–408.0] ms vs. healthy controls: 376.0 [IQR 361.0–393.0] ms; *p* = 0.798) did also lack significant differences compared to the healthy control group. Figure 1 depicts the sFflt-1/PlGF ratio and maternal hemodynamic function in the two cohorts.

### 3.4. Neonatal Outcomes after COVID-19 during Pregnancy

All neonates of pregnant women of both cohorts were liveborn. Neonatal outcome parameters were similar in both groups. The proportion of male neonates was lower in the COVID-19 group (43.5% in the COVID-19 group vs. 67.4% in the healthy control; *p* = 0.097; Table 4).

**Figure 2:**
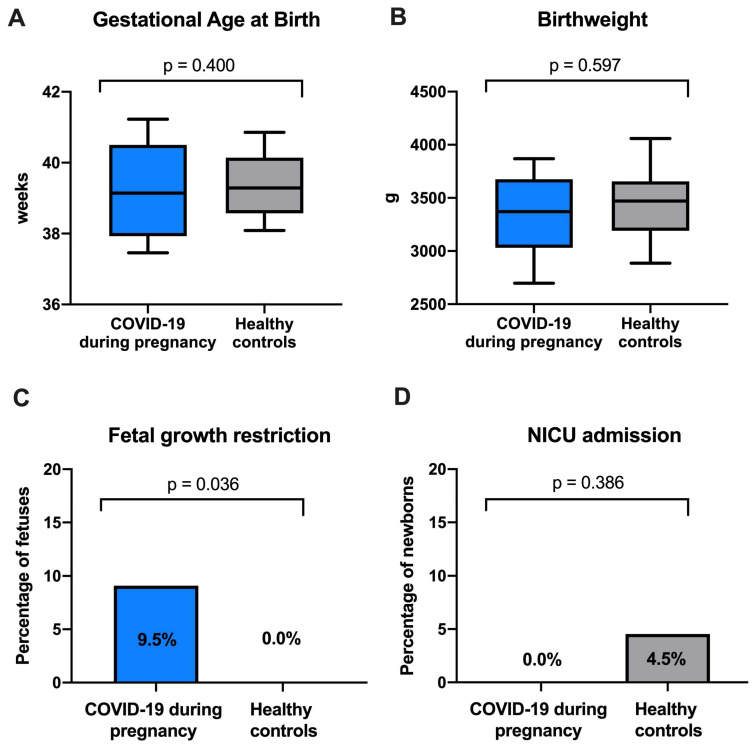
Comparison of (**A**) gestational age at birth, (**B**) birthweight, (**C**) fetal growth restriction and (**D**) newborn intensive care unit (NICU) admission between newborns of women with COVID-19 during pregnancy and healthy controls.

There was no significant difference in median gestational age at birth (COVID-19: 39.0 [IQR 37.5–40.0] weeks vs. healthy controls: 39.0 [IQR 38.0–40.0] weeks; *p* = 0.400) and birthweight (COVID-19: 3370.0 [IQR 3030–3675] g vs. healthy controls: 3470.0 [IQR0. 3190–3655] g; *p* = 0.597). However, importantly, fetal growth restriction (FGR; COVID-19: 9.5% vs. healthy controls: 0.0%; *p* = 0.036) was significantly more frequent in the COVID-19 during pregnancy cohort. Two newborns (n = 2/44; 4.5%) in the healthy control cohort were admitted to the newborn intensive care unit (NICU) both due to RDS versus none in the COVID-19 during pregnancy cohort (n = 0/16; 0.0%; *p* = 0.386). Figure 2 depicts the comparison of neonatal outcome between the two cohorts.

### 3.5. Influence of Parity

There was no difference in age, BMI or gestational age at the time of hemodynamic assessment between nulliparous women in the COVID-19 during pregnancy cohort and in the healthy control cohort (Table S1). Nulliparous women with COVID-19 during pregnancy did not have significantly different CO (5.9 [IQR 5.7–8.0] L/min vs. healthy controls: 6.7 [IQR 5.9–7.2] L/min; *p* = 0.999) and SVR (1062.0 [763.0–1117.0] d.s.cm^−5^ vs. healthy controls: 1034.0 [907.0–1125.0]; *p* = 0.999) as compared to healthy controls. The prevalence of FGR was significantly higher among nulliparous women with COVID-19 during pregnancy (20.0% vs. healthy controls: 0.0%; *p* = 0.027).

## 4. Discussion

### 4.1. Summary of the Main Study Findings

This study compared angiogenic markers and hemodynamic measurements of pregnant women with COVID-19 during pregnancy with healthy pregnant controls. Pregnant women with COVID-19 included in this study all showed relatively mild courses of the disease with only two women requiring hospital admission and no women needing non-invasive or invasive ventilation. Importantly, there was no significant difference in median serum sFlt-1/PlGF levels, CO, SVR and PVWao, suggesting a mostly unchanged hemodynamic status after mild COVID-19 during pregnancy. However, stroke work, the work that the heart has to perform at every contraction [25], was significantly higher in the COVID-19 during pregnancy group.

There were no significant differences observed in GA at birth, as well as other neonatal parameters including APGAR score 5 min after birth, NICU admission and stillbirth did not differ between the COVID-19 during pregnancy and the healthy control group. However, there was a significantly higher prevalence of FGR in the COVID-19 during pregnancy group.

### 4.2. Interpretation of Study Findings and Comparison with Published Literature

The pregnant body undergoes a number of physiological hemodynamic changes during pregnancy, such as a reduction in peripheral vascular resistance and blood pressure as well as an increase in blood volume and cardiac output [26]. Existing data show a change in these hemodynamic alterations in pregnant individuals who develop conditions such as hypertensive disorders of pregnancy [27].

At the same time, there is evidence that SARS-CoV-2 infection during pregnancy is associated with a change in various cardiac markers, such as troponin and BNP, particularly in severe courses of the disease [14,15,16]. Furthermore, it has been shown that pregnant individuals with COVID-19 during pregnancy exhibit an increased risk for the development of preeclampsia [17], which has been found to be independent of preexisting conditions and COVID-19 severity [18]. This suggests that SARS-CoV-2 infection during pregnancy might lead to long-term changes of maternal hemodynamic function which could be associated with higher risk of preeclampsia. In our cohort of pregnant women with COVID-19, there was no significant difference in angiogenic markers or hemodynamic parameters such as CO, SVR and PWVao compared to a healthy control group. This may be attributed to the mild course of COVID-19 in our cohort. Our data suggest that mild COVID-19 does not lead to significant changes in hemodynamic function of pregnant women. However, FGR tended to be more prevalent among women with COVID-19 during pregnancy. This is in line with previous data showing a higher risk of FGR after COVID-19 during pregnancy [28,29]. While an association between COVID-19, preterm birth and stillbirth was described by previous studies [30], this could not be observed in our study cohort.

Previous studies have shown that nulliparous women are at particular risk of developing preeclampsia after COVID-19 during pregnancy [18]. In line with this, we observed more frequent development of FGR among nulliparous women with COVID-19 during pregnancy. We therefore suggest that nulliparous women with COVID-19 during pregnancy should be closely monitored for the development of FGR in pregnancy.

### 4.3. Strengths and Limitations

This is the first study to thoroughly investigate maternal hemodynamic alterations in women with COVID-19 during pregnancy. For this, we used two non-invasive devices: continuous wave Doppler ultrasound and oscillometric arterial stiffness measurement. By comparing the hemodynamic parameters as well as angiogenic markers and clinical parameters of women with COVID-19 during pregnancy to a healthy control cohort, we were able to assess different aspects of SARS-CoV-2 infection during pregnancy.

This study has some limitations: Firstly, this is a monocentric study and thus requires external validation. Secondly, the sample size is limited, potentially masking minor changes in maternal hemodynamic parameters after COVID-19 during pregnancy. Thirdly, the included women presented with relatively mild COVID-19 courses. One can speculate that hemodynamic alterations after COVID-19 might be more pronounced with severe courses of the disease; however, further research is required to support this hypothesis. Furthermore, sFlt-1/PlGF levels were normal among the women in the COVID-19 during pregnancy cohort of this study, which represents an important difference to other studies, which reported an increased risk of preeclampsia after COVID-19 [17,18]. This might also be attributed to the fact that only mild COVID-19 courses were included in this study. Additionally, the vaccination status of included women with COVID-19 during pregnancy has not been assessed. Due to the exploratory nature of the study, no post hoc corrections for multiple comparisons were performed.

## 5. Conclusions

Our study showed that pregnant women with mild courses of COVID-19 did not present with significant differences in maternal hemodynamic function or angiogenic marker levels compared to a healthy control group. However, women with COVID-19 during pregnancy might be at higher risk for developing FGR, although numbers were small in our cohort.

## Figures and Tables

**Table 1:** Patient characteristics of women participating in this study. Data presented as mean ± standard deviation (SD) or median with interquartile range (IQR), as appropriate, or numbers (%).

	COVID-19 during Pregnancy (n = 23)	Healthy Controls (n = 46)	*p*-Value
Maternal age, years (IQR)	32.2 (28.8–35.7)	31.9 (29.3–35.6)	0.932
Maternal BMI, kg/m^2^	24.7 (23.5–33.6)	24.1 (21.9–26.9)	0.268
Gestational age at hemodynamic measurement, weeks ± SD	25.5 ± 5.6	24.0 ± 5.9	0.324
Nulliparous, n (%)	11 (47.83%)	23 (50.0%)	0.865
Mode of conception			0.123
Spontaneous, n (%)	20 (87.0%)	44 (95.7%)	
In vitro fertilization, n (%)	1 (4.3%)	2 (4.3%)	
Intracytoplasmic sperm injection (%)	2 (8.7%)	0 (0.0%)	
Cardiovascular disease, n (%)	2 (8.7%)	0 (0.0%)	0.042
Lung disease, n (%)	1 (4.3%)	0 (0.0%)	0.154
Diabetes, n (%)	1 (4.3%)	0 (0.0%)	0.154
Hematological disease, n (%)	0 (0.0%)	0 (0.0%)	-
Liver disease, n (%)	0 (0.0%)	0 (0.0%)	-
Endocrinological disease, n (%)	2 (8.7%)	0 (0.0%)	0.042
Neurological disease, n (%)	2 (8.7%)	0 (0.0%)	0.042
Renal disease, n (%)	1 (4.3%)	0 (0.0%)	0.154
Smoking during pregnancy	0 (0.0%)	1 (3.0%)	0.400

**Table 2:** COVID-19 symptoms and outcomes of included patients with SARS-CoV-2 infection during pregnancy. Data presented as median and IQR or numbers (%).

	COVID-19 during Pregnancy
**Symptoms**	
Headache, n/n Total (%)	11/17 (64.7%)
Chills, n/n Total (%)	6/17 (35.3%)
Fever, n/n Total (%)	7/17 (41.2%)
Duration of fever, days (IQR)	3.0 (1.0–19.0)
Sneeze, n/n Total (%)	7/17 (41.2%)
Rhinitis, n/n Total (%)	12/17 (70.6%)
Loss of smell/taste, n/n Total (%)	13/17 (76.5%)
Sore throat, n/n Total (%)	9/17 (52.9%)
Cough, n/n Total (%)	12/17 (70.6%)
Dyspnea, n/n Total (%)	8/17 (47.1%)
Conjunctivitis, n/n Total (%)	1/16 (6.3%)
Arthralgia, n/n Total (%)	6/17 (35.3%)
Myalgia, n/n Total (%)	8/17 (47.1%)
Vertigo/fatigue, n/n Total (%)	11/17 (64.7%)
Nausea, n/n Total (%)	8/17 (47.1%)
Emesis, n/n Total (%)	5/17 (29.4%)
Diarrhea, n/n Total (%)	4/17 (23.5%)
**Outcomes**	
Hospital admission, n/n Total (%)	2/23 (8.7%)
Intensive care unit admission, n/n Total (%)	0/23 (0.0%)
Non-invasive ventilation, n/n Total (%)	0/23 (0.0%)
Intubation, n/n Total (%)	0/23 (0.0%)

**Table 3:** Comparison of sFlt-1, PlGF and their ratio as well as parameters of maternal hemodynamic function between patients with SARS-CoV-2 infection during pregnancy and healthy controls. Data presented as median and IQR or numbers (%).

	COVID-19 during Pregnancy (n = 23)	Healthy Controls (n = 46)	*p*-Value
sFlt-1/PlGF ratio, median (IQR)	4.3 (2.6–5.8)	3.6 (1.8–6.3)	0.795
sFlt-1, pg/mL (IQR)	1543.5 (1076.5–2022.5)	1422.5 (1133.0–1756.0)	0.795
PlGF, pg/mL (IQR)	350.5 (234.0–518.5)	422.5 (242.0–627.0)	0.437
**USCOM-1A^®^ Monitor**			
Body Surface Area, m^2^ (IQR)	1.9 (1.8–2.2)	1.8 (1.8–1.9)	0.551
Outflow Tract Diameter (OTD), (IQR)	1.9 (1.9–1.9)	1.9 (1.9–2.0)	0.670
Mean Arterial Pressure, mmHg (IQR)	83.0 (79.0–95.0)	82.5 (79.0–87.5)	0.544
Velocity Peak, median (IQR)	1.5 (1.4–1.6)	1.5 (1.4–1.6)	0.648
Heart Rate, beats/min (IQR)	80.0 (72.0–87.0)	75.0 (69.0–82.0)	0.443
Stroke Volume (SV), mL (IQR)	92.0 (81.0–103.0)	87.0 (73.0–96.0)	0.268
Stroke Volume Index (SVI), mL/m^2^ (IQR)	46.0 (40.0–54.0)	48.0 (39.0–53.5)	0.669
Cardiac Output (CO), L/min median (IQR)	7.1 (5.9–8.2)	6.7 (5.8–7.2)	0.348
Cardiac Index, L/min/m^2^ median (IQR)	3.6 (3.2–4.1)	3.6 (3.1–4.0)	0.797
Systemic Vascular resistance (SVR), d.s.cm^−5^ (IQR)	998.0 (890.0–1087.0)	1030.0 (921.0–1125.5)	0.670
Systemic Vascular Resistance Index (SVRI), d.s.cm^−5^ m^2^ (IQR)	1861.0 (1665.0–2082.0)	1896.0 (1634.0–2241.0)	0.932
Stroke Volume Variability, median (IQR)	14.0 (12.0–20.0)	11.0 (7.3–20.5)	0.074
Inotropy Index, W/m^2^ (IQR)	1.6 (1.5–1.8)	1.8 (1.5–1.8)	0.670
PE/KE Ratio, median (IQR)	23.0 (17.0–28.0)	22.0 (20.0–26.0)	0.932
Stroke work, mmHg/mL (IQR)	1060.0 (901.0–1161.0)	926.5 (812.0–1072.0)	**0.033**
Cardiac Power, W (IQR)	1.3 (1.1–1.7)	1.2 (1.0–1.3)	0.106
**Arteriograph**			
Pulse Pressure, mmHg (IQR)	47.0 (42.0–54.0)	47.0 (42.0–53.0)	0.826
Brachial Augmentation index, % (IQR)	−52.9 (−60.9–−45.6)	−61.1 (−67.6–−44.7)	0.924
Central systolic Blood Pressure (SBPao), mmHg (IQR)	100.0 (94.0–108.0)	100.0 (92.0–111.0)	0.942
Central aortic Pulse Pressure (Ppao), mmHg (IQR)	35.2 (30.4–36.0)	34.3 (31.1–39.8)	0.942
Augmentation index Aortic, % (IQR)	10.9 (6.8–14.5)	6.7 (3.5–15.0)	0.942
Aortic Pulse Wave Velocity (PWVao), m/s (IQR)	7.3 (6.5–8.4)	7.1 (6.4–7.9)	0.504

**Table 4:** Comparison of neonatal outcomes of newborns of patients with SARS-CoV-2 infection during pregnancy and of healthy controls. Data presented as median and IQR or as number (%).

	COVID-19 during Pregnancy (n = 23)	Healthy Controls (n = 46)	*p*-Value
Sex			0.097
Male, n (%)	10 (43.5%)	31 (67.4%)	
Female, n (%)	11 (47.8%)	14 (30.4%)	
Unknown, n (%)	2 (9.5%)	1 (2.2%)	
Gestational age at birth, weeks (IQR)	39.0 (37.5–40.0)	39.0 (38.0–40.0)	0.400
Birthweight, g (IQR)	3370.0 (3030.0–3675.0)	3470.0 (3190.0–3655.0)	0.597
FGR, n/n Total (%)	2/21 (9.5%)	0/45 (0.0%)	0.036
Mode of delivery			0.226
vaginal delivery, n (%)	9 (39.1%)	28 (60.9%)	
ventouse, n (%)	3 (13.0%)	2 (4.3%)	
elective cesarean section, n (%)	1 (4.4%)	0 (0.0%)	
primary cesarean section, n (%)	4 (17.4%)	10 (21.7%)	
secondary cesarean section, n (%)	4 (17.4%)	5 (10.9%)	
Unknown, n (%)	2 (8.7%)	1 (2.2%)	
APGAR score < 7 5 min after birth, n (%)	0 (0.0%)	0 (0.0%)	-
NICU admission, n/n Total (%)	0/16 (0.0%)	2/44 (4.5%)	0.386
Stillbirth, n (%)	0 (0.0%)	0 (0.0%)	-

## Data Availability

The data are available upon reasonable request to the corresponding author.

## Supplementary Materials

The following supporting information can be downloaded at: https://www.mdpi.com/article/10.3390/v16060868/s1. Table S1. Comparison of preeclampsia markers, hemodynamic parameters and neonatal outcomes of pregnant women SARS-CoV-2 infection stratified for parity.
