# Supplementary material for: The Impact of COVID-19 during Pregnancy on Maternal Hemodynamic Function, Angiogenic Markers and Neonatal Outcome
Source: Viruses. 2024 May 29;16(6):868. doi: 10.3390/v16060868 (PMC11209264; doi:10.3390/v16060868)
Supplement: Supplementary file 1 [file viruses-16-00868-s001.zip › viruses-2971491-supplementary.pdf]

SUPPLEMENTARY MATERIAL

The impact of COVID-19 during pregnancy on maternal hemodynamic function, angiogenic markers and neonatal outcome

Nawa SCHIRWANI-HARTL[1], Lena TSCHANUN[1], Pilar PALMRICH[1], Christina HABERL[1], Nicole PERKMANN-NAGELE[2], Herbert KISS[1], Angelika BERGER[3], Julia BINDER[1]

[1]Department of Obstetrics and Gynecology, Division of Obstetrics and feto-maternal Medicine, Medical University of Vienna, Vienna, Austria

[2]Department of Laboratory Medicine, Medical University of Vienna, Vienna, Austria

[3]Department of Pediatrics and Adolescent Medicine, Division of Neonatology, Pediatric Intensive Care and Neuropediatrics, Comprehensive Center for Pediatrics, Medical University of Vienna, Vienna, Austria

SHORT TITLE: COVID-19 during pregnancy and hemodynamics

Table S1. Comparison of preeclampsia markers, hemodynamic parameters and neonatal outcomes of pregnant women SARS-CoV-2 infection stratified for parity.
Data presented as median and IQR or as number (%)

	COVID-19 during pregnancy (n=11)	Healthy controls (n=23)	p-value
Age, years (IQR)	31.7 (26.1-36.7)	31.6 (29.5-33.4)	0.999
BMI, kg/m^2 (IQR)	24.3 (19.4-29.1)	24.0 (21.6-26.5)	0.999
Gestational age at hemodynamic measurement, weeks (IQR)	25.6 (21.6-29.3)	28.3 (20.1-29.0)	0.463
sFLT-1/PlGF, median (IQR)	5.3 (3.6-8.2)	3.6 (2.3-4.8)	0.463
Mean arterial pressure, mmHg (IQR)	79.0 (76.0-82.0)	83.0 (79.0-90.0)	0.143
Stroke Volume (SV), mL (IQR)	98.0 (76.0-103.0)	86.0 (71.0-96.0)	0.999
Stroke Volume Index (SVI), mL/m^2 (IQR)	44.0 (42.0-56.0)	48.0 (40.0-52.0)	0.999
Cardiac Output (CO), L/min median (IQR)	5.9 (5.7-8.0)	6.7 (5.9-7.2)	0.999
Cardiac Index, L/min/m^2 median (IQR)	3.4 (3.1-3.9)	3.6 (3.1-4.0)	0.999
Systemic Vascular resistance (SVR), d.s.cm^{-5} (IQR)	1062.0 (763.0-1117.0)	1034.0 (907.0-1125.0)	0.999
Systemic Vascular Resistance Index (SVRI), d.s.cm^{-5}m^2 (IQR)	1830.0 (1630.0-1964.0)	1926.0 (1636.0-2165.0)	0.463
Brachial Augmentation index, % (IQR)	-53.1 (-64.0 - -34.9)	-64.2 (-73.2 - -52.2)	0.605

Central systolic Blood Pressure (SBPao), mmHg (IQR)	97.0 (89.7-112.5)	103.2 (94.0-111.7)	0.761
Aortic Augmentation index, % (IQR)	10.8 (5.3-20.0)	5.1 (0.6-11.2)	0.605
Aortic Pulse Wave Velocity (PWVao), m/s (IQR)	7.9 (6.4-9.3)	7.4 (6.4-8.2)	0.926
Gestational age at birth, weeks (IQR)	39.1 (38.0-40.5)	39.6 (38.4-40.3)	0.729
Birthweight, g (IQR)	3370.0 (2850.0-3667.5)	3352.5 (3047.5-3641.3)	0.908
Length at birth, cm (IQR)	49.0 (48.5-52.5)	51.0 (50.0-53.5)	0.999
Head circumference, cm (IQR)	35.5 (32.5-36.3)	35.0 (34.0-35.8)	0.225
FGR, n/n Total (%)	2/10 (20.0%)	0/23 (0.0%)	**0.027**
Mode of birth			0.471
vaginal delivery, n (%)	4 (36.4%)	16 (69.6%)	
ventouse, n (%)	2 (18.2%)	2 (8.7%)	
elective cesarean section, n (%)	0 (0.0%)	0 (0.0%)	
primary cesarean section, n (%)	1 (9.0%)	2 (8.7%)	
secondary cesarean section, n (%)	2 (18.2%)	2 (8.7%)	
Unknown, n (%)	2 (18.2%)	1 (4.3%)	
APGAR score <7 5 minutes after birth, n (%)	0 (0.0%)	0 (0.0%)	-
